# Effects of Dietary Nitrate Supplementation on Physiological Responses, Cognitive Function, and Exercise Performance at Moderate and Very-High Simulated Altitude

**DOI:** 10.3389/fphys.2017.00401

**Published:** 2017-06-09

**Authors:** Oliver M. Shannon, Lauren Duckworth, Matthew J. Barlow, Kevin Deighton, Jamie Matu, Emily L. Williams, David Woods, Long Xie, Blossom C. M. Stephan, Mario Siervo, John P. O'Hara

**Affiliations:** ^1^Research Institute for Sport, Physical Activity, and Leisure, Leeds Beckett UniversityLeeds, United Kingdom; ^2^Defence Medical Services, Royal Centre for Defence MedicineBirmingham, United Kingdom; ^3^Institute for Ageing and Health, Newcastle UniversityNewcastle upon Tyne, United Kingdom

**Keywords:** beetroot juice, nitrate, nitric oxide, altitude, exercise performance

## Abstract

**Purpose:** Nitric oxide (NO) bioavailability is reduced during acute altitude exposure, contributing toward the decline in physiological and cognitive function in this environment. This study evaluated the effects of nitrate (${\text{NO}}_{3}^{-}$) supplementation on NO bioavailability, physiological and cognitive function, and exercise performance at moderate and very-high simulated altitude.

**Methods:**Ten males (mean (SD): $\dot{\text{V}}{\text{O}}_{2\text{max}}$: 60.9 (10.1) ml·kg^−1^·min^−1^) rested and performed exercise twice at moderate (~14.0% O_2_; ~3,000 m) and twice at very-high (~11.7% O_2_; ~4,300 m) simulated altitude. Participants ingested either 140 ml concentrated ${\text{NO}}_{3}^{-}$-rich (BRJ; ~12.5 mmol ${\text{NO}}_{3}^{-}$) or ${\text{NO}}_{3}^{-}$-deplete (PLA; 0.01 mmol ${\text{NO}}_{3}^{-}$) beetroot juice 2 h before each trial. Participants rested for 45 min in normobaric hypoxia prior to completing an exercise task. Exercise comprised a 45 min walk at 30% $\dot{\text{V}}{\text{O}}_{2\text{max}}$ and a 3 km time-trial (TT), both conducted on a treadmill at a 10% gradient whilst carrying a 10 kg backpack to simulate altitude hiking. Plasma nitrite concentration ([${\text{NO}}_{2}^{-}$]), peripheral oxygen saturation (SpO_2_), pulmonary oxygen uptake ($\dot{\text{V}}{\text{O}}_{2}$), muscle and cerebral oxygenation, and cognitive function were measured throughout.

**Results:** Pre-exercise plasma [${\text{NO}}_{2}^{-}$] was significantly elevated in BRJ compared with PLA (*p* = 0.001). Pulmonary $\dot{\text{V}}{\text{O}}_{2}$ was reduced (*p* = 0.020), and SpO_2_ was elevated (*p* = 0.005) during steady-state exercise in BRJ compared with PLA, with similar effects at both altitudes. BRJ supplementation enhanced 3 km TT performance relative to PLA by 3.8% [1,653.9 (261.3) vs. 1718.7 (213.0) s] and 4.2% [1,809.8 (262.0) vs. 1,889.1 (203.9) s] at 3,000 and 4,300 m, respectively (*p* = 0.019). Oxygenation of the gastrocnemius was elevated during the TT consequent to BRJ (*p* = 0.011). The number of false alarms during the Rapid Visual Information Processing Task tended to be lower with BRJ compared with PLA prior to altitude exposure (*p* = 0.056). Performance in all other cognitive tasks did not differ significantly between BRJ and PLA at any measurement point (*p* ≥ 0.141).

**Conclusion:** This study suggests that BRJ improves physiological function and exercise performance, but not cognitive function, at simulated moderate and very-high altitude.

## Introduction

Exposure to altitude, an environment where the partial pressure of oxygen (PO_2_) is reduced relative to sea-level, has a profound negative effect on physiological function and exercise performance (Bärtsch and Saltin, 2008). Arterial oxygen saturation declines (Calbet and Lundby, 2009), muscle metabolism is perturbed (Richardson et al., 2006; Vanhatalo et al., 2011), and maximal oxygen consumption ($\dot{\text{V}}{\text{O}}_{2\text{max}}$) is decreased (Wehrlin and Hallén, 2006; MacInnis et al., 2015). Accordingly, exercise time to exhaustion (TTE) is reduced, and time-trial (TT) performance is slower at altitude compared with sea-level (Fulco et al., 1998). Interventions which help attenuate the decline in physiological functioning and exercise performance at altitude are therefore highly desirable. One strategy which has attracted considerable recent attention in this regard is dietary nitrate (${\text{NO}}_{3}^{-}$) supplementation.

Dietary nitrate (${\text{NO}}_{3}^{-}$) is an inorganic anion that can be reduced into nitrite (${\text{NO}}_{2}^{-}$) and subsequently nitric oxide (NO) in the body. Elevating NO bioavailability via ${\text{NO}}_{3}^{-}$ supplementation has been reported to restore muscle metabolic function and leg-extension TTE to normoxic levels during exposure to moderate simulated altitude (i.e., normobaric hypoxia; fraction of inspired oxygen (F_I_O_2_): 14.5%; ~2,800 m) (Vanhatalo et al., 2011). Others have also observed increased peripheral oxygen saturation (SpO_2_) (Masschelein et al., 2012; Muggeridge et al., 2014; Bourdillon et al., 2015; Shannon et al., 2016), elevated muscle oxygenation (Masschelein et al., 2012), improved endothelial function (Bakker et al., 2015), and enhancements in high-intensity TTE (Vanhatalo et al., 2011; Masschelein et al., 2012; Kelly et al., 2014) and TT performance (Muggeridge et al., 2014; Shannon et al., 2016) across a range of altitudes/ simulated altitudes (F_I_O_2_: 11–15%; 2,500–5,000 m) consequent to ${\text{NO}}_{3}^{-}$ supplementation. Interestingly, one study reported that ${\text{NO}}_{3}^{-}$ supplementation was more effective in normobaric hypoxia (F_I_O_2_ 13.1%; ~3,700 m) compared with normoxia (Kelly et al., 2014). Indeed, ${\text{NO}}_{3}^{-}$ supplementation reduced oxygen consumption and enhanced severe-intensity cycle ergometry TTE in hypoxia, but not in normoxia (Kelly et al., 2014). As the reduction of ${\text{NO}}_{2}^{-}$ into NO is potentiated as oxygen tension declines (Castello et al., 2006), it is reasonable to suggest that the effects of ${\text{NO}}_{3}^{-}$ supplementation on NO bioavailability, signaling, and physiological responses might also increase with the degree of hypoxia. Thus, ${\text{NO}}_{3}^{-}$ supplementation may be most effective at very-high compared with moderate altitudes, although this remains to be determined.

Previous studies exploring the performance effects of ${\text{NO}}_{3}^{-}$ supplementation at altitude/simulated altitude have employed high-intensity leg-extension (Vanhatalo et al., 2011), cycle ergometry (Masschelein et al., 2012; Kelly et al., 2014; Muggeridge et al., 2014; Bourdillon et al., 2015; MacLeod et al., 2015) or treadmill running (Arnold et al., 2015; Shannon et al., 2016) as an exercise modality. However, exercise at altitude, particularly hiking, or mountaineering, often involves prolonged bouts of lower-intensity activity (Mellor et al., 2014). The effects of ${\text{NO}}_{3}^{-}$ supplementation on key physiological or functional (e.g., time required to walk a given distance) parameters during this type of activity are presently unclear. However, further study is warranted given a relatively small number of people ascending to altitude are trained athletes conducting high-intensity exercise, yet thousands of individuals undertake altitude hiking and mountaineering each year (Shah et al., 2015).

Several studies conducted at sea-level indicate that ${\text{NO}}_{3}^{-}$ supplementation might enhance cognitive function. An early study in type II diabetics reported improved simple reaction time following ${\text{NO}}_{3}^{-}$ supplementation (Gilchrist et al., 2014). More recently, studies have reported elevated cerebral blood flow and improved response accuracy during the Serial 3 Subtraction Task in healthy individuals (Wightman et al., 2015), and improved response time to cognitive tests at rest (Thompson et al., 2016) and during prolonged intermittent exercise (Thompson et al., 2015) in team sport players supplemented with dietary ${\text{NO}}_{3}^{-}$. The effect of ${\text{NO}}_{3}^{-}$ on cognitive function at altitude is an attractive area for exploration, given cognitive function is typically compromised at altitude, and this may have negative safety implications for individuals hiking or mountaineering in this environment (Abraini et al., 1998; Li et al., 2000; Taylor et al., 2016). To the authors' knowledge, only one study has been conducted in this area to date. Lefferts et al. (2016) reported no effects of a low dose of ${\text{NO}}_{3}^{-}$ (~5 mmol) on resting cognitive function in conditions of very-high simulated altitude (F_I_O_2_: 11.6%; ~4,600 m). Nevertheless, it remains to be established whether a higher dose of ${\text{NO}}_{3}^{-}$ might be necessary to enhance cognitive function at altitude. Further, it is unclear whether the effects of ${\text{NO}}_{3}^{-}$ on cognitive function might vary between different altitudes, or whether ${\text{NO}}_{3}^{-}$ supplementation might elicit different effects when conducted during hypoxic exercise compared to rest. Further study is therefore warranted.

Against this background, the purpose of the present study was to evaluate the effects of ${\text{NO}}_{3}^{-}$ supplementation on physiological and cognitive function, and exercise performance at moderate (3,000 m) and very-high (4,300 m) simulated altitude.

## Methods

### Participants

Ten healthy men with a mean (SD) age of 23 (3) years, body mass of 78.0 (12.5) kg, stature of 180.3 (8.1) cm, and sea-level maximal rate of oxygen uptake ($\dot{\text{V}}{\text{O}}_{2\text{max}}$) of 60.9 (10.1) ml·kg^−1^·min^−1^ volunteered and provided fully informed written consent to participate in the present study. All participants were non-smokers, normotensive and not currently taking any medication. None of the participants had traveled to an altitude >1,500 m during the preceding 3 months and all were currently residing <500 m. The study received institutional ethical approval and adhered to the principles of the Declaration of Helsinki.

### Overview

Participants attended the laboratory on five separate occasions within a 7 week period. Visits were separated by a minimum of five and maximum of 12 days, and conducted at the same time each day (±1 h) to minimize the influence of circadian variance. On the first laboratory visit, participants completed an incremental exercise test to volitional exhaustion in normoxia to elucidate $\dot{\text{V}}{\text{O}}_{2\text{max}}$. Following a rest period of ~20 min, participants were then familiarized with the experimental procedures. All subsequent visits involved exercise in a normobaric hypoxic chamber (Sporting Edge, Sherfield on Loddon, UK), situated ~113 m above sea-level. The F_I_O_2_ inside the normobaric hypoxic chamber was adjusted on a daily basis, accounting for fluctuations in barometric pressure and for 47 mmHg water vapor pressure (Conkin, 2011), to simulate either a moderate altitude (F_I_O_2_ ~14.5%; ~3,000 m) or very-high altitude (F_I_O_2_: ~11.7%; ~4,300 m). There were, in total, four different experimental conditions, which were conducted in a randomized order. These were: (1) 3,000 m simulated altitude with ${\text{NO}}_{3}^{-}$-rich beetroot juice (BRJ) supplementation, (2) 3,000 m simulated altitude with ${\text{NO}}_{3}^{-}$-deplete placebo beetroot juice (PLA) supplementation, (3) 4,300 m simulated altitude with BRJ supplementation; and, (4) 4,300 m simulated altitude with PLA supplementation. Prior to the experimental trials, participants consumed either 140 ml concentrated BRJ (~12.5 mmol ${\text{NO}}_{3}^{-}$) or PLA (~0.01 mmol ${\text{NO}}_{3}^{-}$) (Beet It, James White Ltd., Ipswich, UK) administered double blind, 2 h before arriving at the laboratory. The ${\text{NO}}_{3}^{-}$ content of supplements was determined via ozone-based chemiluminescence, as previously described (Shannon et al., 2016). Participants were asked to avoid alcohol and caffeine consumption, and abstain from intense exercise for 24 h prior to each trial. Additionally, participants were asked to avoid antibacterial mouthwash and chewing gum throughout the study duration, given these have previously been demonstrated to destroy the oral nitrate reducing bacteria (Govoni et al., 2008). Participants gave verbal confirmation at each trial that they had fully adhered to the stipulated pre-trial controls.

### Preliminary trials

A two-part incremental test was conducted on a motorized treadmill (Woodway, Cranlea, Birmingham, UK) at sea-level. During the first part of the test, participants completed four submaximal stages of 3 min duration, interspersed with 1 min recovery periods. The treadmill speed was set at 1 km·h^−1^ for the first stage, and increased by 1 km·h^−1^ each subsequent stage. The treadmill gradient was set to 10% for all submaximal stages, and participants were required to carry a 10 kg backpack. Gas data obtained during this first part of the test was used to calculate the $\dot{\text{V}}{\text{O}}_{2}$-speed relationship, and allow determination of the appropriate treadmill speed for submaximal exercise in the experimental trials. The exercise modality has previously been utilized to approximate the physiological demands of altitude hiking (Matu et al., 2017). Following four exercise stages, participants rested for ~5 min, after which the second phase of the test commenced. Participants removed the backpack, and the treadmill gradient was lowered to 1%. Participants were then required to run at a fixed speed, which was determined based around perceived fitness and eliciting an initial rating of perceived exertion (RPE) of ~12. The treadmill gradient was increased by 1% every minute until participants reached volitional exhaustion. Expired gas was monitored continuously throughout exercise via an online gas analysis system, which was calibrated prior to testing in accordance with the manufacturer's instructions (MedGraphics Ultima CPX, MGC Diagnostics, MN, USA). Gas data obtained during the second part of the test was used to determine $\dot{\text{V}}{\text{O}}_{2\text{max}}$ (highest 30-s average in $\dot{\text{V}}{\text{O}}_{2}$). Following a rest period of ~20 min, participants were familiarized with the cognitive testing procedures and the 3 km walk test, as applied in the experimental trials.

### Experimental trials

**Figure 1:**
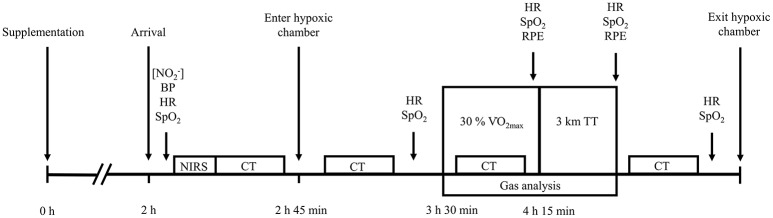
A schematic representation of the experimental trials protocol. [${\text{NO}}_{2}^{-}$], assessment of plasma nitrite concentration; BP, blood pressure; HR, heart rate; SpO_2_, peripheral oxygen saturation; NIRS, near-infrared spectroscopy baseline; CT, cognitive testing; RPE, ratings of perceived exertion; TT, time-trial.

Approximately 1 week after preliminary testing, participants completed the first of four experimental trials. Experimental trials commenced with ~45 min of sitting at sea-level, during which time pre-hypoxic exposure measurements were obtained. Participants then entered the normobaric hypoxic chamber, simulating an altitude of either 3,000 or 4,300 m, where they rested for a further 45 min. An exercise period then commenced, 1.5 h after participant arrival at the laboratory and 3.5 h post-supplementation. Exercise comprised 45 min of steady-state walking at 30% $\dot{\text{V}}{\text{O}}_{2\text{max}}$, followed immediately by a 3 km TT. An exercise intensity of 30% of sea-level $\dot{\text{V}}{\text{O}}_{2\text{max}}$ elicited a walking speed of 2.6 (0.6) km·h^−1^, which replicated the low intensity nature of high altitude hiking (Matu et al., 2017; Mellor et al., in press). During the TT, participants were informed of the distance completed every 500 m, but were blinded to the speed and time. All exercise was conducted at a 10% gradient whilst participants carried a 10 kg backpack. Following exercise participants rested in the normobaric hypoxic chamber for a further 30 min, after which they returned to sea-level. A schematic representation of the experimental trials protocol is presented in Figure 1.

### Measurements

#### Plasma [${\text{NO}}_{2}^{-}$] and blood pressure

On arrival at the laboratory, participants were asked to rest in a seated position for 5 min. An 8 ml venous blood sample was collected via venepuncture from a vein in the antecubital fossa into lithium heparin containing vacutainers (Becton Dickinson, Plymouth, UK). Blood was immediately centrifuged for 3 min at 5,000 rpm. Plasma was subsequently extracted into opaque cryotubes (Argos Technologies, IL, USA), which were pre-treated with 6.5 mM nethylmaleimide (NEM) and 0.1 mM diethylenetriaminepentaacetic acid (DTPA) to minimize the interchange between NO metabolites (Nagababu and Rifkind, 2010). Cryotubes were placed in a freezer at −80˙C, and later analyzed for plasma ${\text{NO}}_{2}^{-}$ concentration ([${\text{NO}}_{2}^{-}$]) via ozone-based chemiluminescence, as previously described (Shannon et al., 2016). After blood sampling, participants rested for a further 5 min. Blood pressure (BP) of the brachial artery was then measured using an automated sphygmomanometer (Omron Healthcare Ltd., Kyoto, Japan). Four measures were acquired, and the mean value of the final three measurements was used for data analysis.

#### Peripheral oxygen saturation and heart rate

Following BP assessments, SpO_2_, and HR were measured via pulse oximetry (Nellcor, Medtronic, Minneapolis, MN). Subsequent measures of SpO_2_ and HR were conducted at rest in hypoxia immediately prior to exercise, during the final 5 min of steady-state walking, immediately following the TT, and prior to exiting the hypoxic chamber.

#### Cognitive function assessment

Cognitive function was assessed at rest prior to hypoxic exposure, at rest in hypoxia, during steady-state walking (after 15 min walking), and 5 min following TT completion. All cognitive assessments were conducted using the Cambridge Neuropsychological Test Automated Battery (CANTAB, Cambridge Cognition, Cambridge, UK), and administered via a touch screen tablet computer (IPad Air, Apple Inc., Cupertino, CA, US). Three tests which have previously been applied to evaluate the efficacy of nutritional supplementation on cognitive function were administered (Maylor et al., 2006; Gilchrist et al., 2014; Thompson et al., 2015; Wightman et al., 2015). These were the Attention Switching Task (AST), Rapid Visual Information Processing Task (RVP), and Spatial Span Task (SST). Tasks were selected to provide information across different cognitive domains including executive function, attention, and working memory capacity.

##### Attention switching task

The AST is a test of executive function that measures the participant's ability to switch attention between stimuli, and ignore task irrelevant information. White arrows are displayed on a black background, with the arrows located on either the left or right side of the screen, and pointing either to the left or to the right. A cue is displayed at the same time as the arrows, reading either “SIDE” or “DIRECTION.” When the “SIDE” cue is presented, the participant is required to press a button on the left or right of the computer screen corresponding to the side of the screen where the arrow is presented, regardless of the direction the arrow is pointing in. Conversely, when the “DIRECTION” cue is presented, the participant is required to touch a button on the left or right of the computer screen corresponding to the direction the arrow is pointing, regardless of which side of the screen the arrow is presented. The task administration time was ~8 min, including congruent stimuli (i.e., the location and direction of the arrow are the same) and incongruent stimuli (i.e., the location and direction of the arrow are different). The total number of correct responses and response times to the test were analyzed.

##### Rapid visual information processing task

The RVP is a test of sustained attention. The participant is presented with a white box in the center of the computer screen. Single digits ranging from 2 to 9 are presented one at a time in a pseudo-random order inside the box, appearing at a rate of 100 digits per minute. The participant is required to detect specific 3-digit sequences, including 2-4-6, 4-6-8, and 3-5-7. As soon as a target sequence is detected, the participant is required to touch a button on the screen. The task administration time was ~10 min. The number target sequences correctly identified, response time for correctly identified sequences, and number of false alarms were analyzed.

##### Spatial span task

The SSP is a visuospatial analog of the Digit Span Task, and measures working memory capacity. The participant is initially presented with a black screen containing 9 white boxes. In a variable sequence, some of the boxes briefly change color. The participant is required to touch the boxes in the same order in which they changed color. Initially, two boxes change color. For every successful correct response, the number of boxes changing color is increased by one, up to a maximum of nine boxes changing color. The sequence and color change of boxes is varied throughout the task. The test terminates when the participant fails to correctly identify the sequence at a particular level on three occasions, or correctly identifies all nine boxes that change color during the final stage of the task. The task administration time was ~5 min. The longest sequence of correctly identified boxes and mean number of attempts to pass were analyzed.

#### Muscle near-infrared spectroscopy

Near Infrared spectroscopy (NIRS) is a popular non-invasive technique for evaluating tissue oxygenation (Neary, 2004). In the present study, a dual wavelength (760 and 850 nm), light weight (84 g), portable NIRS device (Portamon, Artinis Medical Systems, The Netherlands) was used for this purpose. During rest in normoxia, following the measurement of resting plasma [${\text{NO}}_{2}^{-}$], BP, HR, and SpO_2_, a small patch was shaved on the left lateral gastrocnemius. The portable NIRS device was secured to the leg via a combination of surgical tape and an elastic non-compressive bandage. The bandage also served the combined role of shielding the device from extraneous light. The device placement was replicated between trials with reference to local anatomical sites and the small shaved patch on the leg. The device uses the modified Beer-Lambert Law to monitor concentration changes in oxyhaemoglobin ([O_2_Hb]), deoxyhaemoglobin ([HHb]), and total hemoglobin ([tHb]). Values are expressed relative to the first datum, which was collected following 5 min of seated rest in normoxia. The [HHb] signal, which is regarded as being essentially blood volume insensitive during exercise (Blasi et al., 1993), was used to provide an estimate of fractional O_2_ extraction in the area under interrogation (Masschelein et al., 2012; Bailey et al., 2015). The sum of [O_2_Hb]and [HHb] was used to calculate [tHb], which provides an index of the change in regional blood volume (van Beekvelt et al., 2001). This device also measures the Tissue Saturation Index (TSI) using spatially resolved spectroscopy (SRS)—a measure which provides a percentage oxygenation figure that reflects the ratio between absolute values of oxygenated and total hemoglobin plus myoglobin in the area under interrogation. The TSI is calculated as: [O_2_Hb]/([O_2_Hb] + [HHb]) ^*^ 100. Data was recorded at 10 Hz, and a differential pathlength factor (DPF) of 4.94 was used, in accordance with the findings of Duncan et al. (1995). Mean muscle [O_2_Hb], muscle [HHb], and muscle [tHb] were calculated for 5 min prior to and during each cognitive testing period at rest and during exercise, and overall for the 3 km TT.

#### Cerebral near-infrared spectroscopy

Cerebral oxygenation was also monitored via means of a cerebral NIRS device. A dual wavelength (760 and 850 nm), light weight (230 g), portable cerebral NIRS device (Octamon, Artinis Medical Systems, The Netherlands) was used for this purpose. The unit consisted of a headband with 8 light emitters and two light detectors, with an interoptode distance of 3.5 cm. The device placement was replicated between trials by positioning the bottom of the headband 1 cm above the eyebrows, and the middle of the headband in the center of the forehead. This device principally measures oxygenation of the medial prefrontal cortex. As with the muscle tissue NIRS device, the cerebral NIRS applies the modified Beer-Lambert Law to monitor changes in [O_2_Hb], [HHb], and [tHb] relative to the first datum, which was obtained following 5 min of seated rest in normoxia. Data were recorded at 10 Hz and averaged for each variable across all channels to provide an index of pre-frontal cortex oxygenation. The DPF was adjusted according to the participants age (Duncan et al., 1995). Concentration changes in cerebral [O_2_Hb], cerebral [HHb], and cerebral [tHb] were calculated individually during the 5 min period prior to each bout of cognitive tests, during each cognitive task (at rest and during exercise), and overall for the 3 km TT.

#### Pulmonary gas exchange and ratings of perceived exertion

Pulmonary $\dot{\text{V}}{\text{O}}_{2}$, $\dot{\text{V}}{\text{CO}}_{2}$, and respiratory exchange ratio (RER) were monitored throughout exercise, as previously described. Mean $\dot{\text{V}}{\text{O}}_{2}$, $\dot{\text{V}}{\text{CO}}_{2}$, and RER were calculated for a 30 min period during steady-state walking (10–40 min), and for the entire 3 km TT. During the final 5 min steady-state exercise and immediately post-TT, RPE was also recorded.

### Statistical analysis

Data were analyzed using IBM SPSS version 24 for Windows. Two-way (supplement × altitude) repeated measures ANOVA was employed to assess differences in plasma [${\text{NO}}_{2}^{-}$], BP, HR, SpO_2_, RPE, cognitive task performance, pulmonary gas exchange data, muscle [HbO_2_], muscle [HHb], muscle [tHb], and TT performance across experimental conditions. Cerebral [HbO_2_], cerebral [HHb], and cerebral [tHb] were compared during TT via two-way (supplement × altitude) ANOVA, and during each cognitive testing period via three-way (supplement × altitude × task) repeated measures ANOVA. Significant three-way interaction effects were explored using one-way ANOVA and two-way interactions were explored using paired *t*-tests. Statistical significance was set at *p* < 0.05 and Bonferroni corrections were applied to all *post-hoc* tests. Cohen's *d* is presented for information on effect sizes. Effect sizes were interpreted as ≤0.2 trivial, >0.2 small, >0.6 moderate, >1.2 large, >2 very large, and >4 extremely large (Hopkins, 2004).

A statistical spreadsheet was also employed to derive qualitative probabilistic inferences for TT performance data (Hopkins, 2007). Verbal descriptors were used to express the chance that the true value of the effect was beneficial, trivial, or harmful, according to the following scale: <0.5%, “almost certainly not”; 0.5–5%, “very unlikely not”; 5–25%, “unlikely”; 25–75%, “possibly”; 75–95%, “likely”; 95–99.5%, “very likely”; >99.5%, “almost certainly.” The effect was deemed unclear when an odds ratio of benefit to harm of <66 was identified, corresponding to a 25% chance of benefit and 0.5% risk of harm. Data are presented as means (SD) unless otherwise stated. Individual responses are presented within figures where appropriate to allow further exploration of the findings.

Power calculations were conducted using G^*^Power (Faul et al., 2007) to estimate an appropriate sample size. Based on previously published data from our laboratory (Shannon et al., 2016), power analysis indicated that 10 participants would provide >90% power to detect a 3.2% change in TT performance with an alpha level of 0.05.

## Results

All data is presented for *n* = 10, except for the pre-exercise cognitive function data which is presented for *n* = 9 due to missing data.

### Plasma [${\text{NO}}_{2}^{-}$], and blood pressure

**Figure 2:**
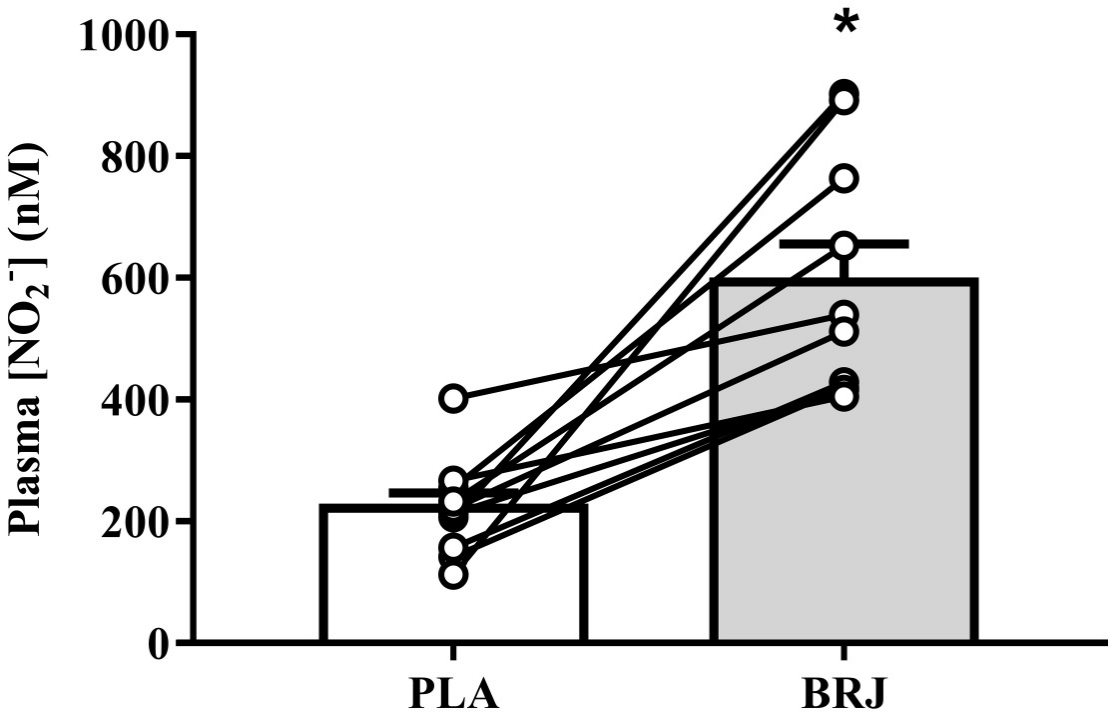
Resting plasma nitrite concentration ([${\text{NO}}_{2}^{-}$]) prior to simulated altitude exposure following nitrate-rich beetroot juice (BRJ) and nitrate-deplete placebo (PLA) supplementation. The closed bar represents the group mean and SEM [${\text{NO}}_{2}^{-}$] from the BRJ trials, and the open bar represents the group mean and SEM [${\text{NO}}_{2}^{-}$] from the PLA trials. Lines represent the individual participant changes in plasma [${\text{NO}}_{2}^{-}$] with BRJ supplementation. ^*^Significant main effect of supplement (*p* < 0.05).

Prior to simulated altitude exposure, plasma [${\text{NO}}_{2}^{-}$] was 630.9 (253.7), 203.9 (70.6), 555.3 (171.9), and 238.3 (125.3) nM for BRJ-3000m, PLA-3000m, BRJ-4300m, and PLA-4300m, respectively. Plasma [${\text{NO}}_{2}^{-}$] was significantly elevated in BRJ compared with PLA (main effect of supplement, *p* = 0.001, *d* = 2.22). There was no difference in plasma [${\text{NO}}_{2}^{-}$] prior to 3,000 and 4,300 m simulated altitude exposure (main effect of altitude, *p* = 0.612, *d* = 0.08). Likewise, there was no supplement × altitude interaction effect for plasma [${\text{NO}}_{2}^{-}$] (*p* = 0.089). Pooled plasma [${\text{NO}}_{2}^{-}$] data for the two BRJ trials and two PLA trials is presented in Figure 2.

Prior to simulated altitude exposure, mean arterial pressure (MAP) was 88 (8), 92 (4), 88 (6), and 92 (11) mmHg for BRJ-3000m, PLA-3000m, BRJ-4300m, and PLA-4300m, respectively. MAP did not differ significantly between supplements (*p* = 0.323, *d* = 0.41) or altitudes (*p* = 0.687, *d* = 0.07). Likewise, there was no supplement × altitude interaction effect for MAP (*p* = 0.912).

### Peripheral oxygen saturation, heart rate, and perceived exertion

Resting SpO_2_ and HR were not significantly different between conditions prior to hypoxic exposure (all effects *p* ≥ 0.110, *d* ≤ 0.26, Table 1). There was a significant main effect of supplement on SpO_2_ during the pre-exercise rest period at simulated altitude and steady-state exercise period (both *p* ≤ 0.017, *d* ≥ 0.28) reflecting an elevated SpO_2_ overall in BRJ compared with PLA at these measurement points. During simulated altitude exposure, SpO_2_ was lower at all measurement points at 4,300 m compared with 3,000 m simulated altitude (main effect of altitude, all *p* ≤ 0.002, *d* ≥ 1.37). There were no supplement × altitude effects at any measurement point (all *p* ≥ 0.127).

**Table 1:** Group mean (SD) peripheral oxygen saturation (SpO_2_) and heart rate (HR) following BRJ and PLA supplementation at 3,000 and 4,300 m simulated altitude.

	**Rest in normoxia**	**Pre-exercise rest in hypoxia**	**Steady-state exercise**	**TT**	**Post-exercise rest in hypoxia**
**SpO**_2_					
BRJ-3000m	98 (1)	91 (5)^*^	84 (2)^*^	77 (6)	88 (4)
PLA-3000m	98 (1)	89 (4)	80 (6)	76 (9)	89 (4)
BRJ-4300m	98 (1)	83 (5)^*^^#^	72 (5)^*^^#^	67 (6)^#^	83 (6)^#^
PLA-4300m	98 (1)	81 (4)^#^	70 (5)^#^	66 (8)^#^	79 (5)^#^
**HR**					
BRJ-3000m	68 (13)	68 (15)	117 (13)	163 (12)	94 (11)
PLA-3000m	61 (9)	70 (13)	116 (7)	154 (12)	91 (8)
BRJ-4300m	67 (13)	72 (13)^#^	124 (9)^#^	158 (5)	87 (15)
PLA-4300m	67 (13)	75 (12)^#^	123 (10)^#^	160 (8)	93 (10)

* *Significantly different from PLA (main effect of supplement, p < 0.05)*.

# *Significantly different from 3,000 m (main effect of altitude, p < 0.05)*.

HR was significantly higher at 4,300 m compared with 3,000 m during the pre-exercise rest period at simulated altitude and steady-state exercise (main effect of altitude, both *p* ≤ 0.026, *d* ≥ 0.38). HR was not significantly different between BRJ and PLA at any measurement point (main effect of supplement, all *p* ≥ 0.132, *d* ≤ 0.32). No supplement × altitude interactions were detected for HR (all *p* ≥ 0.072).

During steady-state exercise, RPE was 10 (2), 10 (2), 12 (1), and 11 (2) for BRJ-3000m, PLA-3000m, BRJ-4300m, and PLA-4300m, respectively. RPE did not differ significantly between supplements (*p* = 0.382, *d* = 0.18), but was significantly greater at 4,300 m compared with 3,000 m (main effect of altitude, *p* = 0.007, *d* = 0.93). There was no supplement × altitude interaction effect (*p* = 1.000). During TT exercise, RPE was 19 (2), 19 (2), 19 (2), and 19 (1) for BRJ-3000m, PLA-3000m, BRJ-4300m, and PLA-4300m, respectively. RPE did not differ significantly between supplements (*p* = 0.604, *d* = 0.06) or altitudes (*p* = 0.678, *d* = 0.06) during the TT. Likewise, there was no supplement × altitude interaction effect for RPE (*p* = 0.879).

### Pulmonary gas exchange

#### Steady-state exercise

During steady-state exercise, mean pulmonary $\dot{\text{V}}{\text{O}}_{2}$ was 17.2 (3.9), 18.1 (3.9), 17.6 (4.0), and 18.5 (4.2) ml·kg^−1^·min^−1^ for BRJ-3000m, PLA-3000m, BRJ-4300m, and PLA-4300m, respectively. Pulmonary $\dot{\text{V}}{\text{O}}_{2}$ was significantly lower in BRJ compared with PLA (main effect of supplement, *p* = 0.020, *d* = 0.21). There was no effect of altitude on $\dot{\text{V}}{\text{O}}_{2}$ during steady-state exercise (*p* = 0.298, *d* = 0.10), and no supplement × altitude interaction was detected (*p* = 0.745).

Pulmonary $\dot{\text{V}}{\text{CO}}_{2}$ during steady-state exercise was 16.4 (3.7), 17.2 (3.1), 17.1 (4.1), and 17.6 (4.0) ml·kg^−1^·min^−1^ for BRJ-3000m, PLA-3000m, BRJ-4300m, and PLA-4300m, respectively. $\dot{\text{V}}{\text{CO}}_{2}$ was significantly lower in BRJ compared with PLA (main effect of supplement, *p* = 0.035, *d* = 0.17), but not significantly different between 3,000 and 4,300 m simulated altitude (main effect of altitude, *p* = 0.364, *d* = 0.11). There was no supplement * altitude interaction effect (*p* = 0.906).

Respiratory exchange ratio (RER) during steady-state exercise was 0.95 (0.05), 0.96 (0.06), 0.97 (0.05), and 0.95 (0.05) BRJ-3000m, PLA-3000m, BRJ-4300m, and PLA-4300m, respectively. RER did not differ significantly between supplements (*p* = 0.592, *d* = 0.10) or altitudes (*p* = 0.835, *d* = 0.10). Likewise, there was no supplement * altitude interaction effect on RER (*p* = 0.542).

#### TT exercise

During TT exercise, mean pulmonary $\dot{\text{V}}{\text{O}}_{2}$ was 34.6 (6.3), 34.1 (5.4), 30.0 (3.9), and 29.7 (5.2) ml·kg^−1^·min^−1^ for BRJ-3000m, PLA-3000m, BRJ-4300m, and PLA-4300m, respectively. $\dot{\text{V}}{\text{O}}_{2}$ was significantly higher during TT exercise at 3000m compared with 4300m simulated altitude (main effect of altitude, *p* < 0.001, *d* = 0.90). However, there was no difference in $\dot{\text{V}}{\text{O}}_{2}$ between BRJ and PLA (main effect of supplement, *p* = 0.229, *d* = 0.09), and no supplement × altitude interaction effect (*p* = 0.579).

Pulmonary $\dot{\text{V}}{\text{CO}}_{2}$ during TT exercise was 36.5 (7.3), 35.7 (6.4), 32.0 (4.4), and 31.6 (5.1) ml·kg^−1^·min^−1^ for BRJ-3000m, PLA-3000m, BRJ-4300m, and PLA-4300m, respectively. $\dot{\text{V}}{\text{CO}}_{2}$ tended to be elevated in BRJ compared with PLA (main effect of supplement, *p* = 0.058, *d* = 0.10), and was significantly greater during exercise at 3,000 m compared with 4,300 m simulated altitude (main effect of altitude, *p* = 0.011, *d* = 0.76). There were no supplement * altitude interaction effect (*p* = 0.745).

Respiratory exchange ratio (RER) during TT exercise was 1.05 (0.04), 1.05 (0.07), 1.06 (0.04), and 1.06 (0.06) for BRJ-3000m, PLA-3000m, BRJ-4300m, and PLA-4300m, respectively, and was no different between supplements (*p* = 0.969, *d* = 0.20) or altitudes (*p* = 0.425, *d* = 0.18). Likewise, there was no supplement ^*^ altitude interaction effect (*p* = 0.967).

### Cognitive function

Data for the cognitive test results is located in the Supplementary data Table 1. There was a tendency toward a reduced number of false alarms in the RVP task in BRJ compared with PLA prior to altitude exposure, although this did not attain statistical significance (main effect of supplement, *p* = 0.056, *d* = 0.30). There was no significant difference in cognitive function for other cognitive tests between BRJ and PLA at all measurement points (main effect of supplement, all *p* ≥ 0.141, *d* ≤ 0.59). There was a significant decline in cognitive function at 4,300 m compared with 3,000 m during steady-state exercise for AST number of correct responses, AST response time, and RVP response time (main effect of altitude, all *p* ≤ 0.041, *d* ≥ 0.32). There was also a significant decline in cognitive function at 4,300 m compared with 3,000 m during the post-exercise measurement period for AST response time, RVP number of correct responses, RVP response time, and RVP false alarms (main effect of altitude, all *p* ≤ 0.016, *d* ≥ 0.48). A tendency toward a reduced number of correct responses at 4,300 m compared with 3,000 m was also detected during the post-exercise period for AST, although this did not attain significance (*p* = 0.053, *d* = 0.51). Significant supplement × altitude interaction effects were detected for AST number of correct responses during steady-state exercise (*p* = 0.032), AST response time during the pre-exercise rest period (*p* = 0.019), and SSP mean number of attempts to pass during the post-exercise rest period (*p* = 0.040). However, none of these achieved statistical significance during *post-hoc* analysis once appropriate adjustments were made for multiple comparisons (*p* ≥ 0.325, *d* ≤ 0.84).

### Muscle near-infrared spectroscopy

**Figure 3:**
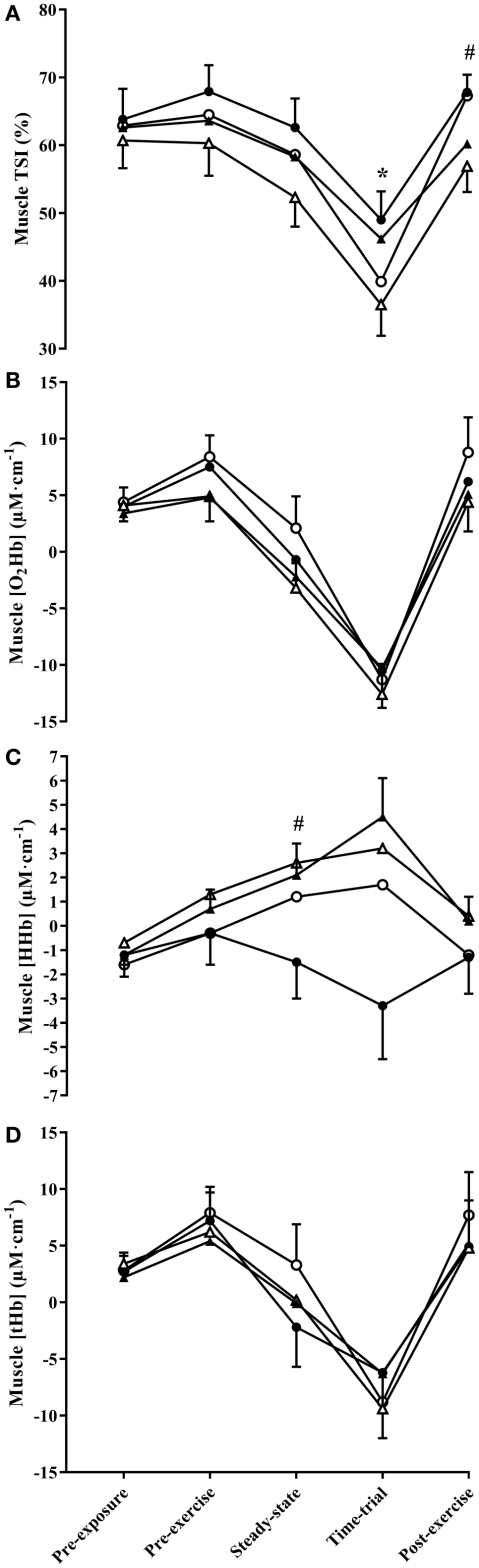
Muscle TSI **(A)**, and the change in muscle oxyhaemoglobin concentration ([HbO_2_]) **(B)**, deoxyhaemoglobin concentration ([HHb]) **(C)**, and total hemoglobin concentration ([tHb]) **(D)** throughout experimental trials at 3,000 and 4,300 m simulated altitude following nitrate-rich beetroot juice (BRJ) and nitrate-deplete placebo (PLA) supplementation. Closed circles, BRJ-3000m; open circles, PLA-3000m; closed triangles, BRJ-4300m; open triangles, PLA-4300m. Data are presented as mean and SEM. ^*^Significant main effect of supplement (*p* < 0.05). ^#^Significant main effect of altitude (*p* < 0.05).

Muscle TSI did not differ significantly between BRJ and PLA during steady-state exercise (main effect of supplement, *p* = 0.139, *d* = 0.15), but was significantly higher in BRJ compared with PLA during TT exercise (main effect of supplement, *p* = 0.011, *d* = 0.71, Figure 3A). In the post-exercise rest period, muscle TSI was significantly greater at 3,000 m compared with 4,300 m (*p* = 0.020, *d* = 0.83). No supplement × altitude interaction effects were observed (*p* ≥ 0.656).

Muscle O_2_Hb did not differ significantly between supplements (all *p* ≥ 0.525, *d* ≤ 0.17, Figure 3B) or altitudes (all *p* ≥ 0.087, *d* ≤ 0.53) at any measurement point. Likewise, there were no supplement × altitude interaction effects (all *p* ≥ 0.205).

Muscle HHb did not differ significantly between BRJ and PLA at any measurement point (all *p* ≥ 0.251, *d* ≤ 0.34, Figure 3C). Muscle HHb was significantly greater at 4,300 m compared with 3,000 m during steady-state exercise (*p* = 0.007, *d* = 0.52), and tended to be greater at 4,300 m compared with 3,000 m during the post-exercise rest period (*p* = 0.051, *d* = 0.38). No significant supplement × altitude interaction effects were observed (all *p* ≥ 0.307).

Muscle tHb did not differ significantly between supplements (all *p* ≥ 0.360, *d* ≤ 0.29, Figure 3D) or altitudes (all *p* ≥ 0.436, *d* ≤ 0.20), and no supplement × altitude interaction effects were detected (all *p* ≥ 0.209) at any measurement point.

### Cerebral NIRS

**Figure 4:**
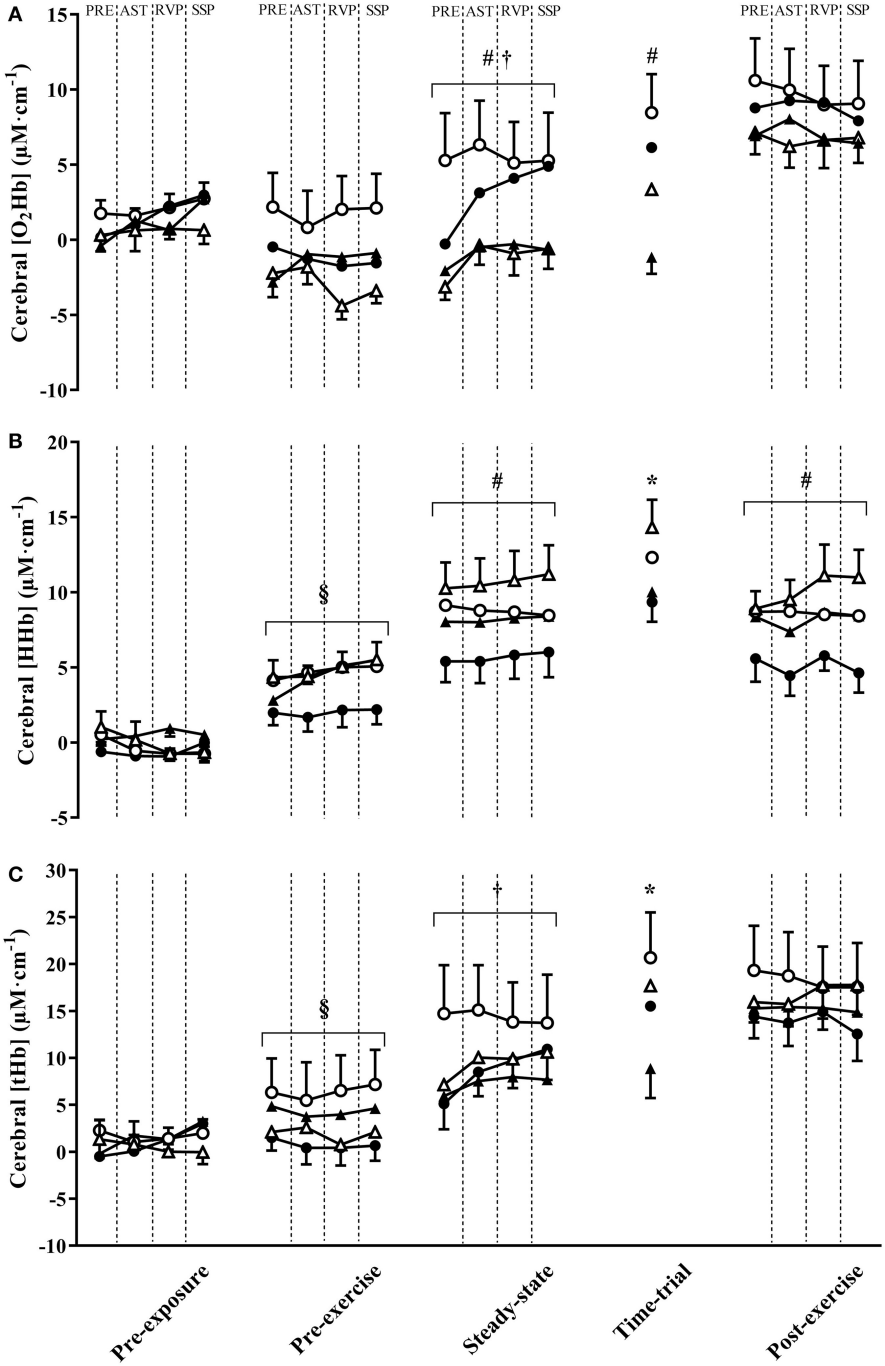
The change in cerebral oxyhaemoglobin concentration ([HbO_2_]) **(A)**, deoxyhaemoglobin concentration ([HHb]) **(B)**, and total hemoglobin concentration ([tHb]) **(C)** throughout experimental trials at 3,000 and 4,300 m simulated altitude following nitrate-rich beetroot juice (BRJ) and nitrate-deplete placebo (PLA) supplementation. Values were obtained in the 5 min prior to cognitive tasks (PRE), and during the Attention Switching Task (AST), Rapid Visual Information Processing Task (RVP), and Spatial Span Task (SSP). Closed circles, BRJ-3000m; open circles, PLA-3000m; closed triangles, BRJ-4300m; open triangles, PLA. Data are presented as mean and SEM. 4300m *Significant main effect of supplement (*p* < 0.05). ^#^Significant main effect of altitude (*p* < 0.05). ^†^Significant main effect of task (*p* < 0.05). ^§^Significant supplement x altitude interaction (*p* < 0.05).

Cerebral O_2_Hb tended to be greater in PLA compared with BRJ during the TT (main effect of supplement, *p* = 0.056, *d* = 0.55, Figure 4A), but did not differ significantly between supplements at other measurement points (all *p* ≥ 0.487, *d* ≤ 0.17). Cerebral O_2_Hb was significantly greater during steady-state and TT exercise at 3,000 m compared with 4,300 m simulated altitude (main effect of altitude, both *p* ≤ 0.001, *d* ≥ 0.88). A significant effect of task was identified during steady-state exercise (*p* < 0.001), reflecting an increase in cerebral O_2_Hb during cognitive tasks compared with values obtained in the 5 min walking period prior to cognitive tasks. A significant supplement × altitude × task interaction effect was also detected during steady-state exercise (*p* = 0.034), however no significant effects were identified during *post-hoc* analysis following adjustments for multiple comparisons (all *p* ≥ 0.909, *d* ≤ 0.71).

Cerebral HHb was significantly greater in PLA compared with BRJ during TT exercise (main effect of supplement, *p* = 0.047, *d* = 0.58, Figure 4B), and tended to be greater during steady-state exercise in PLA compared with BRJ (*p* = 0.053, *d* = 0.53). Cerebral HHb was greater at 4,300 m compared with 3,000 m during steady-state exercise and the post-exercise rest period (both *p* ≤ 0.034, *d* ≥ 0.41). A significant supplement × altitude interaction effect was observed during the pre-exercise rest period (*p* = 0.047). *Post-hoc* analysis revealed greater cerebral HHb in PLA compared with BRJ at 3,000 m during the pre-exercise rest period (*p* < 0.001, *d* = 0.66).

Cerebral tHb was greater in PLA compared with BRJ during TT exercise (main effect of supplement, *p* = 0.043, *d* = 0.64, Figure 4C). There was no difference in cerebral tHb between altitudes (*p* ≥ 0.064, *d* ≤ 0.42). A significant effect of task was identified during steady-state exercise (*p* = 0.016), reflecting the greater tHb during cognitive tasks relative to the 5 min pre-testing walking period. There was also a significant supplement × altitude interaction during the pre-exercise rest period (*p* = 0.021). *Post-hoc* analysis revealed greater tHb in PLA compared with BRJ at 3,000 m simulated altitude during the pre-exercise rest period (*p* < 0.001, *d* = 0.63).

### TT performance

**Figure 5:**
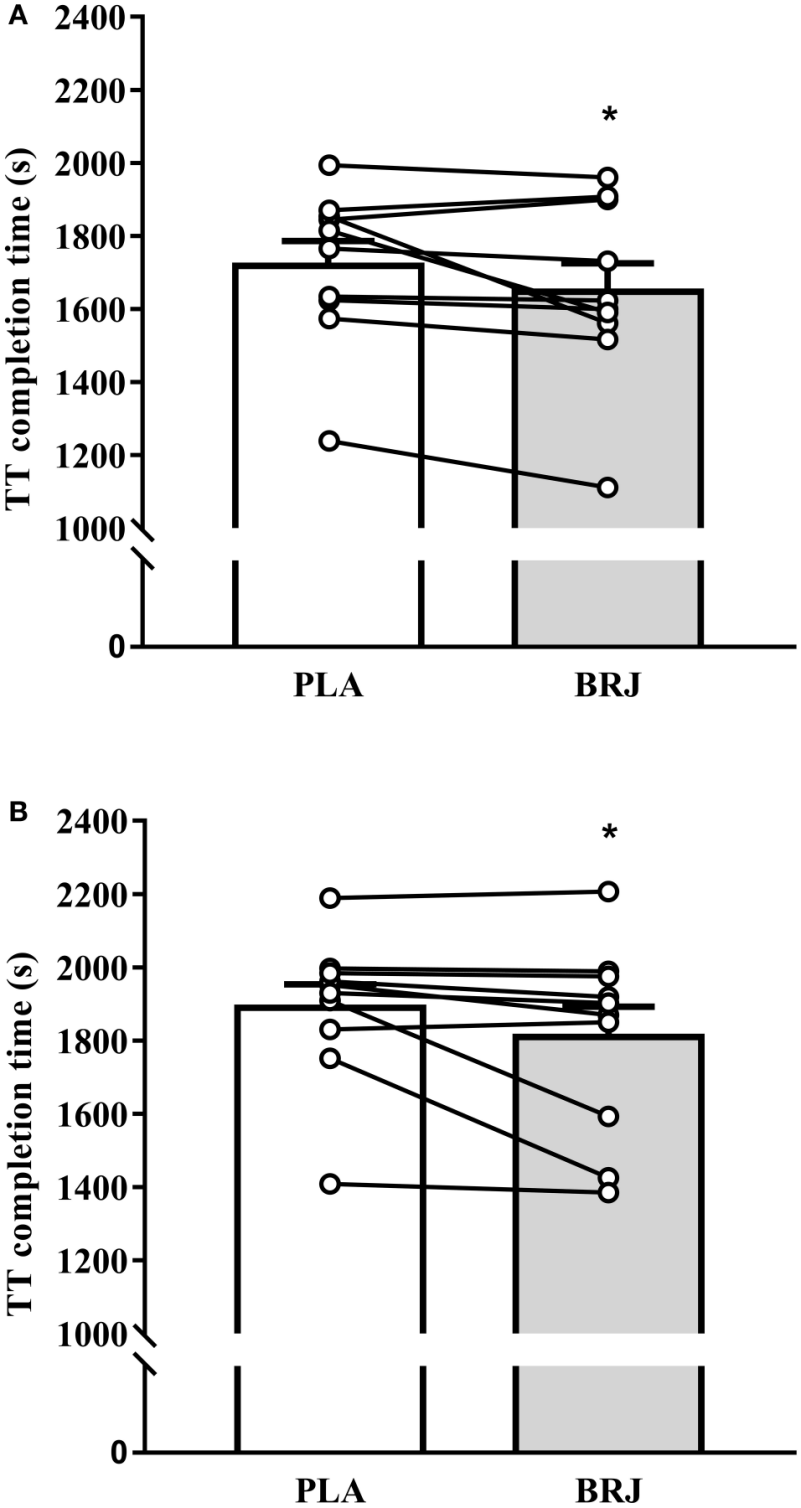
Performance in the 3 km time-trial (TT) at 3,000 m **(A)** and 4,300 m **(B)** simulated altitude following nitrate-rich beetroot juice (BRJ) and nitrate-deplete placebo (PLA) supplementation. The closed bars depicts the mean and SEM performance times from the BRJ trials at each altitude, and the open bar represents the mean and SEM performance times from the PLA trials at each altitude. Lines represent the individual participant changes in performance times with BRJ supplementation. ^*^Significant main effect of supplement (*p* < 0.05).

Completion times for the 3 km TT were 1,653.9 (261.3), 1,718.7 (213.0), 1,809.8 (262.0), and 1,889.1 (203.9) s for BRJ-3000m, PLA-3000m, BRJ-4300m, and PLA-4300m respectively. There was a significant main effect of supplement (*p* = 0.019, *d* = 0.31, Figure 5), reflecting faster TT completion time overall in BRJ compared with PLA. TT completion times were faster at 3,000 m compared with 4,300 m (main effect of altitude, *p* < 0.001, *d* = 0.72). No supplement × altitude interaction effect was detected (*p* = 0.890). Individual threshold values of 42.6 s and 40.8 s were calculated for magnitude-based inferences at 3,000 and 4,300 m, respectively, based around the standardized effect size of 0.2 multiplied by the between subject standard deviation of the placebo trial at each altitude. Magnitude-based inferences indicated a possibly beneficial (74.7%), unlikely trivial (24.9%), most unlikely harmful (0.4%) effect of BRJ on TT performance compared with PLA at 3,000 m. Additionally, the effect of BRJ on TT performance at 4,300 m altitude was deemed likely beneficial (81.2%), unlikely trivial (17.9%), very unlikely harmful (0.9%) compared with PLA.

## Discussion

The present study evaluated the effects of dietary ${\text{NO}}_{3}^{-}$ supplementation on physiological responses, cognitive function, and exercise performance at moderate and very-high simulated altitude. The primary findings are that ${\text{NO}}_{3}^{-}$ supplementation elevated plasma [${\text{NO}}_{2}^{-}$], increased SpO_2_ and muscle TSI, reduced steady-state $\dot{\text{V}}{\text{O}}_{2}$, and improved TT performance. Additionally, ${\text{NO}}_{3}^{-}$ supplementation tended to reduce cerebral [O_2_Hb] and significantly reduced cerebral [HHb] and [tHb] during TT exercise. However, cognitive function was unaffected by ${\text{NO}}_{3}^{-}$ supplementation. Furthermore, the present data do not support the notion that ${\text{NO}}_{3}^{-}$ supplementation is more effective at very-high compared with moderate altitude.

### NO bioavailability and blood pressure

In the present investigation, plasma [${\text{NO}}_{2}^{-}$] was significantly elevated consequent to ${\text{NO}}_{3}^{-}$ supplementation, which substantiates previous research in this area (Webb et al., 2008; Bailey et al., 2009; Vanhatalo et al., 2010, 2011; Wylie et al., 2013; Shannon et al., 2016, 2017). NO generation via L-arginine appears to be blunted in hypoxic conditions (Shaul et al., 1993; Fish et al., 2007). Conversely, the reduction of ${\text{NO}}_{2}^{-}$ into NO is potentiated at low oxygen tensions (Castello et al., 2006). Consequently, the increase plasma [${\text{NO}}_{2}^{-}$] observed following ${\text{NO}}_{3}^{-}$ supplementation may be especially effective at maintaining or enhancing NO bioavailability and signaling in hypoxic conditions, including the moderate and very-high altitude environments simulated in this study.

Elevated NO bioavailability following ${\text{NO}}_{3}^{-}$ supplementation might be expected to lower BP, due to NO-dependent relaxation of smooth muscle and subsequent vasodilation (Larsen et al., 2006; Webb et al., 2008; Vanhatalo et al., 2010; Siervo et al., 2013, 2015). However, in the present study there was no apparent difference in resting MAP assessed prior to simulated altitude exposure between BRJ and PLA. This is also similar to the findings of a recent study by our group, in which trained runners/triathletes showed no change in MAP following administration of an identical ${\text{NO}}_{3}^{-}$ dose, despite substantial increases in plasma [${\text{NO}}_{2}^{-}$] (Shannon et al., 2017). Recently, Ashworth et al. (2015) reported a significant positive relationship between baseline BP and the change in BP following ingestion of a high-${\text{NO}}_{3}^{-}$ diet. Thus, it seems plausible that the already low BP in our participants limited the capacity to further reduce BP following ${\text{NO}}_{3}^{-}$ supplementation.

### Pulmonary gas exchange, peripheral oxygen saturation, and muscle oxygenation

In accordance with multiple previous investigations in both normoxia (Larsen et al., 2007; Bailey et al., 2009; Vanhatalo et al., 2010; Pawlak-Chaouch et al., 2016) and hypoxia (Masschelein et al., 2012; Kelly et al., 2014; Muggeridge et al., 2014; Shannon et al., 2016), ${\text{NO}}_{3}^{-}$ supplementation reduced $\dot{\text{V}}{\text{O}}_{2}$ during steady-state exercise. The novelty in our findings is that we demonstrate this reduction in $\dot{\text{V}}{\text{O}}_{2}$ during simulated altitude hiking, a finding with practical relevance for the thousands of individuals who ascend to altitude for this purpose each year. This was accompanied by an elevated SpO_2_ during steady-state exercise, and a greater muscle TSI during TT exercise consequent to ${\text{NO}}_{3}^{-}$ supplementation. Collectively these data might indicate a reduced tissue oxygen extraction during exercise with ${\text{NO}}_{3}^{-}$ supplementation. ${\text{NO}}_{3}^{-}$ ingestion does not appear to “shift” metabolism toward non-oxidative means of ATP resynthesis (Bailey et al., 2010), and did not alter substrate oxidation in this study as indicated by similar RER values in BRJ and PLA. Therefore, it is likely that these effects reflect a genuine improvement in exercise efficiency (i.e., less oxygen is required for a given work rate). Mechanistically, this could be explained by an improved efficiency of muscle contraction, such that the ATP and oxygen requirements for a particular rate of force generation are reduced (Bailey et al., 2010). Alternatively, ${\text{NO}}_{3}^{-}$ supplementation has been reported to improve the efficiency of mitochondrial ATP resynthesis, an effect likely attributable to reduced proton leak through the ATP/ADP translocase protein (ANT) and possibly uncoupling protein 3 (UCP3), thus lowering the oxygen cost of ATP regeneration (Larsen et al., 2011). However, recent data question whether mitochondrial efficiency is improved with beetroot juice, or whether this effect might be restricted to sodium-${\text{NO}}_{3}^{-}$ supplementation (Whitfield et al., 2015). Contrasting the mechanisms of action of these two ${\text{NO}}_{3}^{-}$ vehicles may be a potential avenue for future research (Kemp, 2016).

Interestingly, in a study by Bourdillon et al. (2015), ${\text{NO}}_{3}^{-}$ supplementation increased pulmonary ventilation and elevated SpO_2_ during exercise in hypoxia. A higher ventilation has previously been associated with increased SpO_2_ (Benoit et al., 1995). Thus, increased ventilation could contribute toward the elevated SpO_2_ with ${\text{NO}}_{3}^{-}$ supplementation in hypoxia. Ventilation-related increases in SpO_2_ might also be expected to increase muscle oxygenation, given an elevated SpO_2_ would increase the pressure gradient driving oxygen diffusion from the blood to the tissue (Masschelein et al., 2012). However, the increased SpO_2_ (during steady-state exercise) and elevated muscle TSI (during TT exercise) occurred during different phases of the exercise test in this study, thus questioning the link between these variables. An acknowledged limitation of this study is that we did not obtain ventilation data, and as such it is unclear whether this parameter was altered following ${\text{NO}}_{3}^{-}$ supplementation.

### Cognitive function and cerebral oxygenation

Some (Gilchrist et al., 2014; Thompson et al., 2015, 2016; Wightman et al., 2015) but not all (Kelly et al., 2013; Bondonno et al., 2014; Thompson et al., 2014) studies conducted at sea-level have reported beneficial effects of ${\text{NO}}_{3}^{-}$ supplementation on cognitive function. Prior to this investigation, to the authors' knowledge only one study had evaluated the effects of ${\text{NO}}_{3}^{-}$ supplementation on cognitive function at simulated altitude (Lefferts et al., 2016). Lefferts et al. (2016) reported a significant decline in short-term memory, information processing efficiency, and emotional recognition during rest at simulated altitude (F_I_O_2_: 11.6%; 4,600 m) compared with sea-level. However, there was no difference in cognitive performance between ${\text{NO}}_{3}^{-}$ and placebo conditions. Likewise, middle cerebral artery blood flow, an index of neurovascular coupling, was unchanged following ${\text{NO}}_{3}^{-}$ supplementation. In the present study, cognitive function was compromised during exercise and the post-exercise rest period at 4,300 m compared with 3,000 m simulated altitude, as indicated by either slower response times or a decreased number of correct responses in the AST and RVP tasks. Resting cognitive function did not differ between supplements at simulated altitude, which is in accordance with the findings of Lefferts et al. (2016). Extending these findings, the present study demonstrated no effect of ${\text{NO}}_{3}^{-}$ supplementation on cognitive function during or following exercise.

The previously reported beneficial effects of ${\text{NO}}_{3}^{-}$ supplementation on cognitive function may be related to alterations in cerebral blood flow, in particular by ensuring optimal delivery of blood (and thus oxygen) to match neural activity (i.e., enhanced neurovascular coupling) (Wightman et al., 2015). In elderly individuals, ${\text{NO}}_{3}^{-}$ supplementation enhanced perfusion to areas of the brain known to play a role in executive function (Presley et al., 2011). Furthermore, ${\text{NO}}_{3}^{-}$ supplementation has been reported to facilitate a faster, smaller, and more homogenous haemodynamic response to visual stimulation (Aamand et al., 2013), and result in an initial increase in cerebral blood flow during cognitive tasks, followed by a consistent reduction in cerebral blood flow during less demanding cognitive tasks (Wightman et al., 2015). In the latter study, these blood flow changes also coincided with improved cognitive performance (Wightman et al., 2015). We observed no difference in pre-frontal cortex [tHb] changes between ${\text{NO}}_{3}^{-}$ and placebo supplementation during the cognitive testing periods. Interestingly, however, ${\text{NO}}_{3}^{-}$ supplementation decreased the change in cerebral [tHb] during the TT, which might be a consequence of lower blood flow to the pre-frontal cortex during the TT in BRJ compared with PLA. Cerebral blood flow changes at altitude are largely determined by four key reflex mechanisms. These are the hypoxic ventilatory response, hypercapnic ventilatory response, hypoxic cerebral vasodilation, and hypocapnic cerebral vasoconstriction (Ainslie and Subudhi, 2014). It is possible that one or more of these variables may have been influenced by ${\text{NO}}_{3}^{-}$ supplementation, with a concomitant effect on cerebral blood flow.

As mentioned, Bourdillon et al. (2015) observed an increase in the hypoxic ventilatory response following ${\text{NO}}_{3}^{-}$ supplementation, which might result in a lower cerebral blood flow by elevating arterial oxygen tensions and decreasing arterial carbon dioxide tensions (Ainslie and Subudhi, 2014). Although we did not assess ventilation in this study, it seems unlikely that a ventilation-related increase in arterial oxygen content accounts for the lower [tHb] in BRJ compared with PLA, given SpO_2_ did not differ between supplements during the TT. A reduction in arterial carbon dioxide tensions could result in cerebral vasoconstriction and hence reduced cerebral blood flow, accounting for the reduced [tHb] during the TT with ${\text{NO}}_{3}^{-}$ supplementation. Arterial carbon dioxide tension was not evaluated in the present study. However, Bourdillon et al. (2015) previously reported no effects of ${\text{NO}}_{3}^{-}$ supplementation on arterial carbon dioxide pressure or end tidal carbon dioxide, which brings into question this mechanism. Alternatively, it is possible that differences in cerebral [tHb] resulted from differences in cerebral activation as a consequence of the higher mean power in BRJ (given time to complete the 3 km TT was lower) compared with PLA (Brümmer et al., 2011).

Cerebral [HHb] was significantly lower during TT exercise, and tended to be lower during steady-state exercise (which involved simultaneous cognitive tasks) following ${\text{NO}}_{3}^{-}$ supplementation. A significant supplement × altitude interaction effect was also observed during the pre-exercise rest period, indicating reduced changes in cerebral [HHb] specifically at 3,000 m simulated altitude consequent to ${\text{NO}}_{3}^{-}$ supplementation. This may indicate a lower cerebral oxygen extraction in the pre-frontal cortex following ${\text{NO}}_{3}^{-}$ supplementation during these measurement periods. Alternatively, as both cerebral [O_2_Hb] and [tHb] were also lower during the TT with BRJ, these findings may reflect a smaller volume of blood in the area under investigation, possibly as a consequence of differences in cerebral blood flow as discussed above. Interestingly, and in contrast to our findings, Masschelein et al. (2012) reported no change in cerebral [O_2_Hb], [HHb], or [tHb] in conditions of extreme simulated altitude (F_I_O_2_ 11%; 5,000 m) with ${\text{NO}}_{3}^{-}$ supplementation. This discordance in findings could be related to the different altitudes simulated in this study and by Masschelein et al. (2012). In hypoxia, cerebral blood flow increases to ensure adequate delivery of oxygen to the brain, with greater changes in blood flow observed at higher compared with lower altitudes (Ainslie and Subudhi, 2014). Thus, it is possible that ${\text{NO}}_{3}^{-}$ may be ineffective at influencing cerebral oxygenation/ blood flow at extreme but not moderate or high altitude, given the comparably greater hyperaemic response in the former.

### TT performance

The effects of ${\text{NO}}_{3}^{-}$ supplementation on exercise TTE and/or TT performance at simulated altitude have been evaluated in a number of previous studies, with some (Vanhatalo et al., 2011; Masschelein et al., 2012; Kelly et al., 2014; Muggeridge et al., 2014; Shannon et al., 2016) but not all (Arnold et al., 2015; Bourdillon et al., 2015; MacLeod et al., 2015) reporting beneficial effects. In contrast to previous studies which have employed high-intensity leg-extension (Vanhatalo et al., 2011), cycle ergometry (Masschelein et al., 2012; Kelly et al., 2014; Muggeridge et al., 2014; Bourdillon et al., 2015; MacLeod et al., 2015), or treadmill running (Arnold et al., 2015; Shannon et al., 2016) exercise protocols at simulated altitude, we evaluated the effects of ${\text{NO}}_{3}^{-}$ supplementation using a protocol which more closely replicates the demands of altitude hiking. For this purpose, our participants were required to complete a 3 km uphill (10% gradient) treadmill test whilst carrying a weighted (10 kg) backpack. We observed a 3.8 and 4.2% improvement in performance in BRJ compared with PLA at 3,000 and 4,300 m simulated altitude, respectively. The effects of BRJ on 3 km TT performance were deemed to be “possibly beneficial” and “likely beneficial” at 3,000 and 4,300 m simulated altitude, respectively (i.e., similar beneficial changes at both simulated altitudes). BRJ supplementation was also deemed “most unlikely harmful” and “very unlikely harmful” (i.e., both <1% chance harmful) for performance at 3,000 and 4,300 m simulated altitude respectively, suggesting adverse performance effects are unlikely with BRJ supplementation. Thus, BRJ supplementation may have potential beneficial applications for individuals hiking at altitude, perhaps in situations where they are required to cover a particular distance quickly, such as in response to changing weather conditions or to ensure completion of a hike within daylight hours.

Several mechanisms have been suggested to explain the beneficial effects of ${\text{NO}}_{3}^{-}$ supplementation on exercise performance. In particular, and as mentioned, ${\text{NO}}_{3}^{-}$ has been proposed to enhance the efficiency of muscle contraction (Bailey et al., 2010) and mitochondrial respiration (Larsen et al., 2011). Such effects might account for the lower $\dot{\text{V}}{\text{O}}_{2}$ and elevated SpO_2_ observed in the present study following ${\text{NO}}_{3}^{-}$ supplementation, and may also be expected to enhance performance by allowing a higher work rate to be maintained for a given $\dot{\text{V}}{\text{O}}_{2}$.

The effects of ${\text{NO}}_{3}^{-}$ supplementation on muscle and cerebral oxygenation might also be relevant from a performance perspective. The greater muscle TSI observed during TT exercise with ${\text{NO}}_{3}^{-}$, reflective of greater muscle oxygenation, could be beneficial by enhancing rates of oxidative ATP resynthesis, and limiting phosphocreatine breakdown and rates of anaerobic glycolysis. This might be beneficial by attenuating the accumulation of fatigue associated metabolites, such as H^+^, P_i_, and ADP (Vanhatalo et al., 2011). Furthermore, it has been suggested that cortical deoxygenation during exercise might limit performance by compromising executive functions and contributing toward the decision to cease exercise, with this effect particularly apparent in hypoxia (Subudhi et al., 2009). It is therefore possible that the beneficial effects of ${\text{NO}}_{3}^{-}$ supplementation on performance might also partly be accounted for by the reduced cerebral deoxygenation during TT exercise.

### Strengths and limitations

The present study makes an important contribution toward our understanding of the applications of ${\text{NO}}_{3}^{-}$ supplementation for individuals exercising at altitude. Notably, our data indicate potential physiological and performance benefits of ${\text{NO}}_{3}^{-}$ supplementation during hiking, which is a popular form of activity amongst individuals ascending to altitude. It is particularly promising that these effects occurred following acute ${\text{NO}}_{3}^{-}$ consumption, such that individuals ascending to altitude may not require prolonged time consuming and costly supplementation strategies to elicit potentially beneficial effects. Nevertheless, certain limitations should be acknowledged.

Firstly, our research was conducted in a normobaric hypoxic chamber which simulated the low PO_2_ of altitude by reducing the F_I_O_2_. This exercise environment allowed greater control over potentially confounding variables vs. testing at terrestrial altitude, and helped minimize participant burden. Recent work has also reported broadly similar responses to normobaric hypoxia and terrestrial altitude (Woods et al., 2017), suggesting that normobaric hypoxia is a reasonable surrogate for the genuine high-altitude environment. Nevertheless, further research is warranted to confirm whether the beneficial effects of ${\text{NO}}_{3}^{-}$ supplementation observed in the present investigation also manifest at terrestrial altitude. It is noteworthy that one recent study reported improvements in flow mediated dilation (FMD) following ${\text{NO}}_{3}^{-}$ ingestion during a trek to 3,700 m terrestrial altitude (Bakker et al., 2015), suggesting the potential capacity to alter some physiological parameters at genuine high altitude with this supplement. The relatively modest sample size of the present study may also be regarded as a limitation. However, our number of participants is similar to several previous investigations that have reported significant beneficial effects of ${\text{NO}}_{3}^{-}$ supplementation (e.g., Bailey et al., 2009; Lansley et al., 2011; Breese et al., 2013; Wylie et al., 2013).

## Conclusion

Relative to placebo, ${\text{NO}}_{3}^{-}$ supplementation reduced steady-state $\dot{\text{V}}{\text{O}}_{2}$, increased peripheral and muscle oxygenation, and improved TT performance during hiking type activity at moderate and very-high simulated altitude. ${\text{NO}}_{3}^{-}$ supplementation also elicited changes to cerebral blood oxygenation, although no differences in cognitive function were observed compared with placebo. These findings suggest that ${\text{NO}}_{3}^{-}$ supplementation may offer some beneficial physiological effects for individuals conducting hiking type activity at moderate and very-high altitude. Conversely, the current data do not support a beneficial effect of ${\text{NO}}_{3}^{-}$ supplementation on cognitive function at altitude.

## Ethics statement

This study was carried out in accordance with the recommendations of the Leeds Beckett University Research Ethics Committee, and adhered to the principles set out in the Declaration of Helsinki. The protocol was approved by the Leeds Beckett University Research Ethics committee. All subjects gave written informed consent prior to participation.

## Author contributions

OS conceived the study. OS, LD, MB, KD, JM, EW, DW, LX, BS, MS, and JO designed the study. OS and JM performed the data collection. LX and MS performed chemiluminescent analysis. OS and KD conducted the statistical analysis. OS, JM, and KD drafted the manuscript. OS, LD, MB, KD, JM, EW, DW, LX, BS, MS, and JO contributed in the revision of the manuscript. All authors approved the final version of the manuscript.

### Conflict of interest statement

The authors declare that the research was conducted in the absence of any commercial or financial relationships that could be construed as a potential conflict of interest.
