# Supplementary material for: Effects of Dietary Nitrate Supplementation on Physiological Responses, Cognitive Function, and Exercise Performance at Moderate and Very-High Simulated Altitude
Source: Front Physiol. 2017 Jun 9;8:401. doi: 10.3389/fphys.2017.00401 (PMC5465306; doi:10.3389/fphys.2017.00401)
Supplement: Supplementary file 1 [file Table1.pdf]

Supplementary data Table 1. Cognitive test results at 3000 m and 4300 m simulated altitude following BRJ and PLA supplementation

					Significance (p) values		
	**BRJ-3000m**	**PLA-3000m**	**BRJ-4300m**	**PLA-4300m**	**Main effect of supplement**	**Main effect of altitude**	**Supplement x altitude interaction**
**AST: Number of correct responses**							
Pre-exposure	154 (5)	155 (6)	154 (5)	155 (4)	0.587	0.733	0.633
Pre-exercise	154 (4)	151 (8)	153 (6)	152 (5)	0.427	0.724	0.342
Steady-state	152 (6)	154 (6)	152 (6)	151 (6)	0.942	0.041*	0.032*
Post-exercise	152 (6)	153 (5)	148 (10)	150 (5)	0.542	0.053	0.709
**AST: Response time (ms)**							
Pre-exposure	476.3 (54.8)	474.7 (64.0)	487.2 (64.5)	480.7 (66.6)	0.749	0.440	0.832
Pre-exercise	462.8 (32.9)	481.4 (56.6)	487.7 (49.9)	460.9 (49.4)	0.818	0.833	0.019*
Steady-state	484.5 (74.9)	486.8 (62.5)	512.9 (69.5)	521.8 (56.5)	0.695	0.021*	0.820
Post-exercise	466.1 (79.0)	469.8 (79.6)	514.5 (92.7)	503.0 (94.4)	0.769	0.013*	0.599
**RVP: Number of sequences correctly identified**							
Pre-exposure	48 (3)	48 (4)	46 (8)	48 (4)	0.628	0.436	0.383
Pre-exercise	47 (6)	45 (8)	45 (8)	47 (7)	0.943	0.850	0.062
Steady-state	49 (4)	48 (7)	47 (5)	47 (6)	0.409	0.138	0.834
Post-exercise	46 (6)	47 (7)	42 (7)	38 (7)	0.368	0.011*	0.176
**RVP: Response time (ms)**							
Pre-exposure	448.8 (84.5)	429.4 (57.0)	445.1 (117.0)	440.9 (115.6)	0.329	0.794	0.605
Pre-exercise	416.8 (40.5)	436.4 (66.8)	422.1 (69.5)	415.1 (69.4)	0.608	0.474	0.064
Steady-state	422.2 (58.6)	424.3 (69.4)	454.5 (82.6)	460.3 (96.0)	0.612	0.025*	0.923
Post-exercise	391.7 (36.4)	383.7 (53.3)	440.5 (60.3)	455.4 (84.4)	0.793	0.000*	0.496
**RVP: Number of false alarms**							
Pre-exposure	2 (2)	4 (4)	3 (3)	3 (2)	0.056	0.904	0.596
Pre-exercise	2 (2)	4 (2)	5 (5)	3 (3)	0.879	0.251	0.073
Steady-state	4 (3)	4 (5)	6 (8)	5 (4)	0.448	0.321	0.596
Post-exercise	4 (3)	3 (2)	5 (3)	7 (3)	0.560	0.016*	0.088

**SSP: Longest sequence correctly identified**							
Pre-exposure	8 (1)	8 (1)	8 (2)	8 (2)	0.269	0.055	0.555
Pre-exercise	9 (1)	8 (1)	9 (1)	8 (1)	0.195	0.342	0.483
Steady-state	8 (1)	8 (1)	8 (1)	8 (1)	0.452	0.104	0.758
Post-exercise	9 (1)	8 (1)	8 (1)	8 (2)	0.684	0.098	0.141
**SSP: Mean number of attempts to pass**							
Pre-exposure	1 (0)	1 (0)	1 (0)	1 (0)	0.169	1.000	1.000
Pre-exercise	1 (0)	1 (0)	1 (0)	1 (0)	0.347	0.347	0.316
Steady-state	1 (0)	1 (0)	1 (0)	1 (0)	0.141	0.415	0.415
Post-exercise	1 (0)	1 (0)	1 (0)	1 (0)	0.794	0.504	0.040*

Data is presented as mean (SD) for n = 10 for pre-exposure, steady-state and post-exercise measurement periods, and n = 9 for the pre-exercise measurement period. * denotes $p < 0.05$.
